# A Tether for Woronin Body Inheritance Is Associated with Evolutionary Variation in Organelle Positioning

**DOI:** 10.1371/journal.pgen.1000521

**Published:** 2009-06-19

**Authors:** Seng Kah Ng, Fangfang Liu, Julian Lai, Wilson Low, Gregory Jedd

**Affiliations:** Temasek Life Sciences Laboratory and Department of Biological Sciences, National University of Singapore, Singapore; Washington University School of Medicine, United States of America

## Abstract

Eukaryotic organelles evolve to support the lifestyle of evolutionarily related organisms. In the fungi, filamentous Ascomycetes possess dense-core organelles called Woronin bodies (WBs). These organelles originate from peroxisomes and perform an adaptive function to seal septal pores in response to cellular wounding. Here, we identify Leashin, an organellar tether required for WB inheritance, and associate it with evolutionary variation in the subcellular pattern of WB distribution. In Neurospora, the *leashin* (*lah*) locus encodes two related adjacent genes. N-terminal sequences of LAH-1 bind WBs via the WB–specific membrane protein WSC, and C-terminal sequences are required for WB inheritance by cell cortex association. LAH-2 is localized to the hyphal apex and septal pore rim and plays a role in colonial growth. In most species, WBs are tethered directly to the pore rim, however, Neurospora and relatives have evolved a delocalized pattern of cortex association. Using a new method for the construction of chromosomally encoded fusion proteins, marker fusion tagging (MFT), we show that a LAH-1/LAH-2 fusion can reproduce the ancestral pattern in Neurospora. Our results identify the link between the WB and cell cortex and suggest that splitting of *leashin* played a key role in the adaptive evolution of organelle localization.

## Author Summary

In the kingdom Fungi, tubular cells called hyphae grow by tip extension and lateral branching to produce an interconnected multicellular syncytium and this unique cellular architecture is especially suited to foraging, long distance transport, and invasive growth. Major groups of fungi have independently evolved cellular organelles that support this form of multicellularity. Woronin bodies evolved over 400 million years ago in the common ancestor of filamentous Ascomycetes and perform an adaptive function to seal pores that connect hyphal compartments (septal pores) in response to cellular wounding. This study identifies Leashin, a tethering protein that promotes equitable Woronin body inheritance by providing a link to the cell cortex. Patterns of cortex association display systematic variation; in most of the filamentous Ascomycetes, Woronin bodies are tethered to the septal pore. By contrast, a delocalized pattern has recently evolved in a group represented by Neurospora and Sordaria. We present evidence suggesting that the ancestral *leashin* gene was split into two independent transcription units to permit this evolutionary transition. This work is exemplary of how filamentous Ascomycetes with well-resolved phylogenetic relationships, diverse sequenced genomes and powerful haploid genetics provide model systems for understanding evolutionary innovation within a functional cellular and physiological context.

## Introduction

Membrane bound organelles are fundamental constituents of eukaryotic cells that execute wide-ranging functions associated with growth and development. Some organelle functions are ubiquitous while others are only found in evolutionarily related organisms and perform lifestyle supporting adaptive functions. Most of the fungi proliferate through the extension and branching of tubular cells called hyphae. Hyphae can be divided into compartments by cell walls known as septa and septal pores provide a connection that allows adjacent cellular compartments to cooperate and coordinate their activities. This syncytial cellular architecture underlies many unique aspects of the fungal lifestyle including rapid radial growth, the invasive growth of saprobes and pathogens and the development of multi-cellular reproductive structures [1]. Major groups of filamentous Basidiomycetes and Ascomycetes have evolved distinct septal pore associated organelles [2]–[4]. Filamentous Ascomycetes (The Pezizomycotina) are a monophyletic group estimated to comprise 90% of Ascomycetes and 50% of all fungal species [5] and these ecologically diverse fungi [6],[7] possess peroxisome-derived organelles called Woronin bodies (WBs) [4],[8].

WBs are centered on a self-assembled matrix protein, HEX, and function to seal the septal pore in response to hyphal wounding [9]–[12]. WB biogenesis occurs in the growing apical hyphal compartment through a process determined in part by apically biased *hex* gene expression [13]. In apical compartments newly synthesized HEX is imported into peroxisomes via its consensus PTS1 sorting signal and assembled *de novo* into micrometer scale protein complexes [13],[14]. The Woronin sorting complex protein (WSC) envelops HEX assemblies to help them bud from the peroxisome matrix [14]. Through physical association with the cell cortex, these newly formed organelles are inherited into sub-apical compartments where they are immobilized and poised to execute their function in pore sealing [13],[15]. Cortex association also requires WSC [14], but the link between WSC and the cell cortex remains unknown.

Organelle Inheritance by cortex association is a recurring theme in fungal systems [16]. In the yeast *Saccharomyces cerevisiae*, organelles need to be equitably partitioned between mother and daughter cells and various organelles share a common strategy that balances cytoskeleton dependent transport into the bud with immobilization at the mother cell cortex. This type of mechanism has been implicated in the segregation of mitochondria [17],[18], peroxisomes [19], and the endoplasmic reticulum [20]. The yeast peroxisome provides an especially clear example; here the peripheral peroxisome membrane protein Inp1 promotes cortex association. Deletion of Inp1 results in excessive acto-myosin dependent peroxisome transport into daughter cells while overproduction results in aberrant immobilization and accumulation of peroxisomes at the mother cell cortex [21],[22]. Interestingly, the ability of WSC to promote cortex association also depends on its level in the membrane [14], suggesting that the accumulation of key membrane proteins may regulate the segregation of diverse organelles.

Within the Pezizomycotina, patterns of WB distribution vary systematically; in most species, WBs are tethered to the septal pore at a distance of 100 nm–200 nm and associate with the pore rim through a filamentous [15] and elastic [23] tether of unknown composition. By contrast, in a group defined by Neurospora and Sordaria, WBs occur at the cell cortex in a delocalized pattern, suggesting that a new pattern evolved in the common ancestor of these genera.

## Results

**Figure 1:**
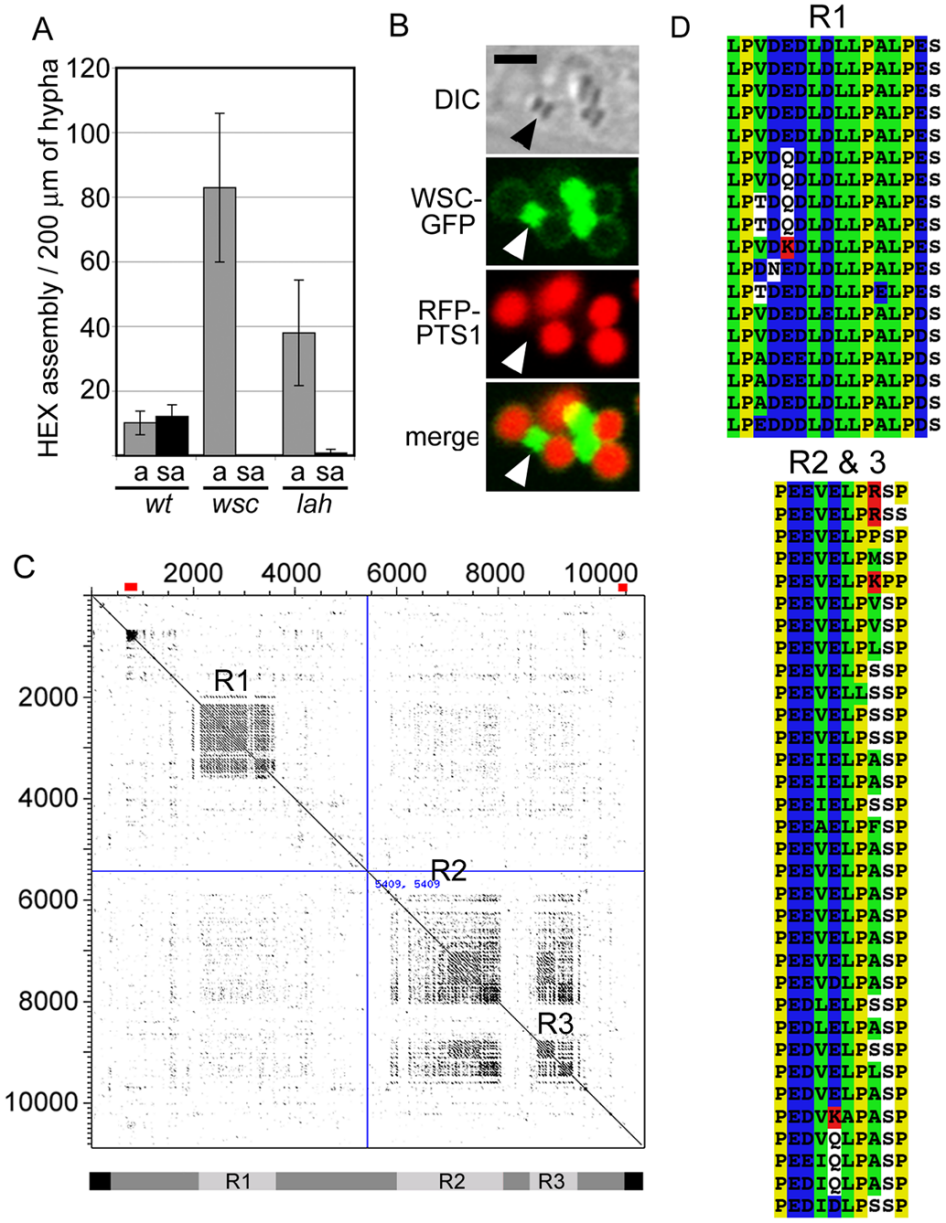
The *leashin* mutant is defective in Woronin body inheritance. (A) The *lah* mutant accumulates HEX assemblies in the apical compartment. The distribution of HEX assemblies was quantified in apical (a) and sub-apical (sa) hyphal compartments in the indicated strains. (B) HEX assemblies are enveloped by WSC in the *lah* mutant background. RFP-PTS1 reveals the peroxisome matrix and WSC-GFP reveals assembly of the sorting complex. HEX assemblies can be seen in the DIC channel. Arrowhead points to a pair of aberrantly associated nascent WBs. Bar = 2 µm. (C) Dot plots reveal repetitive sequences R1, R2 and R3 in the predicted LAH polypeptide. The schematic indicates conserved regions (black bars) and repeat regions (light gray bars). Red bars indicate two predicted coiled-coil domains. (D) Alignment of repetitive sequences using Clustal W [44]. R1 contains 18 repeats centered on a core consensus sequence, LPVDEDLDLLPALPES, and R2 and R3 contain 33 repeats of the consensus sequence, PEEVELPASP. Acidic residues are colored blue, basic residues are red, hydrophobic sequences are green and Proline is indicated in yellow.

In a screen for genes involved in WB segregation, we identified a spontaneous mutation that behaves as a single recessive locus and accumulates HEX assemblies in the apical compartment (Figure 1A) and based on our functional analysis, we named this locus *leashin* (*lah*). To assess the effect of the *lah* mutation on the function of WSC, the *lah* mutant was transformed to express WSC-GFP and RFP-PTS1 (A marker of the peroxisome matrix). In the *lah* background, WSC envelops HEX assemblies to produce budding intermediates similar to those observed in wild-type cells [14], however, these accumulate and aggregate aberrantly (Figure 1B) in the apical hyphal compartment, suggesting that LAH functions downstream of WSC and plays a role in WB inheritance.

*lah* was mapped to the left arm of chromosome I by meiotic recombination and one cosmid carrying an ∼30-kilobase gene, NCU02793, was found to complement the *lah* WB segregation defect and NCU02793 deletion results in phenotypes similar to those observed in the original *lah* mutant (data not shown). The *lah* gene encodes the largest predicted protein (10,821 amino acids) found in the Neurospora genome and homologs were identified in all sequenced genomes of the Pezizomycotina; these predicted proteins range in size from the smallest from *Aspergillus nidulans* (5,936 amino-acids) to the largest in *Giberella zea* (7,480 amino-acids). Overall these proteins exhibit complex N- and C- terminal sequences separated by poorly conserved intervening sequences (Figure S1). Predicted Neurospora *leashin* is highly acidic with a calculated charge of −1088 at neutral pH and encodes three repetitive regions, R1, R2, and R3 (Figure 1C and 1D), which are enriched in proline, leucine and acidic amino acids aspartic acid and glutamic acid. Below, we present evidence showing that Neurospora *leashin* actually comprises two transcription units encoding distinct proteins, which we name Leashin-1 and Leashin-2.

**Figure 2:**
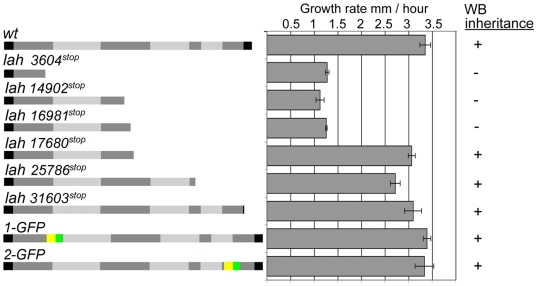
3′-sequences of *lah* are dispensable for WB–associated functions. Growth rate and WB–segregation were assessed in the indicated strains. Truncations were produced at the indicated positions by integrating stop codons to the chromosome using the *hyg^r^* marker under control of the *trpC* promoter. The last two cartoons depicted epitope tagged versions of *lah* produced by marker fusion tagging. Yellow = *hyg^r^*, green = GFP.

To begin to dissect the function of *leashin*, we used homologous recombination to introduce a stop codon at various positions of the predicted *lah* gene. Numbering from the start codon, truncation at nucleotide positions 3604, 14902 and 16981, produce defects in WB inheritance. By contrast, truncations at 17680, 25786 and 31603 do not interfere with WB segregation and cause mild defects in maximal hyphal growth rate (Figure 2). These data indicate that the 5′-half of the *lah* locus is required for WB segregation while 3′-regions are dispensable.

**Figure 3:**
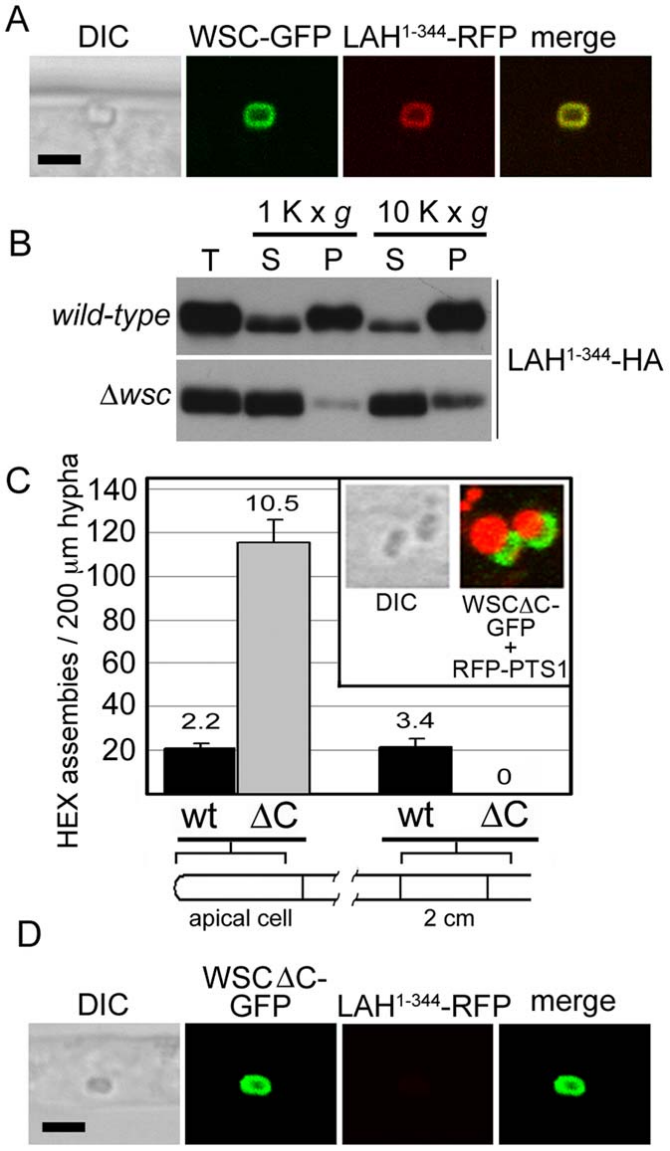
N-terminal sequences of LAH associate with WBs via C-terminal sequences of WSC. (A) The first 344 amino acids of LAH co-localize with WSC at the WB surface. LAH^1–344^-RFP was co-expressed with WSC-GFP and visualized by confocal microscopy. Bar = 2 µm. (B) LAH^1–344^ association with a dense organellar fraction depends on the presence of WSC. LAH^1–344^-HA was expressed in wild-type and *wsc* mutant background and a crude organellar fraction (T) was separated into supernatant (S) and pellet (P) fractions and the distribution of LAH^1–344^-HA was revealed with anti-HA epitope antibodies. (C) C-terminal tail of WSC is required for WB-segregation but not the production of nascent WBs. HEX assembly distribution was assessed in a strain expressing WSCΔC-GFP. Inset shows the distribution of WSCΔC-GFP and RFP-PTS1, which reveals the peroxisome matrix. (D) LAH^1–344^ does not associate with the WB in the WSCΔC-GFP expressing strain. WSCΔC-GFP and LAH^1–344^-RFP were co-expressed and visualized as in (A). Bar = 2 µm.

LAH should localize to the WB surface if it plays a direct role in WB segregation. We next assessed localization of LAH fragments of fused to the red fluorescent protein (RFP) and identified an N-terminal domain encompassing amino acids 1–344 (LAH^1–344^RFP) that co-localizes with WSC-GFP at the WB surface (Figure 3A). In extracts prepared from cells expressing an HA-epitope tagged version of this LAH fragment, the fusion protein sediments at very low centrifugal forces, consistent with association with the dense-core WB, but is rendered mostly soluble in extracts prepared from a *wsc* deletion strain (Figure 3B), suggesting that LAH associates with WBs via WSC. To further investigate this interaction, we examined WSC deletion mutants; deletion of the WSC C-terminal tail (Δ236–307) blocks WB segregation, but not the envelopment of HEX assemblies and production of nascent WBs (Figure 3C). WB localization of LAH^1–344^RFP is also abolished in the WSC C-terminal deletion, further suggesting that N-terminal sequences of LAH associate with WBs through the C-terminus of WSC (Figure 3D).

The large size of the predicted *lah* gene precluded its manipulation by standard recombinant methods. This prompted us to develop a new method, marker fusion tagging (MFT) that allows tagging and deletion analysis of chromosomally encoded genes. Briefly, fusion PCR was used to generate an in-frame fusion between the dominant selectable marker for Hygromycin resistance (*hyg^r^*) and eGFP or the 3×HA epitope tag. A second round of fusion PCR was then used to append in-frame fragments from *leashin* to this central cassette. The integration of this cassette by homologous recombination results in the production of a fusion protein where Hygromycin resistance is engendered by the chromosomally encoded fusion protein.

**Figure 4:**
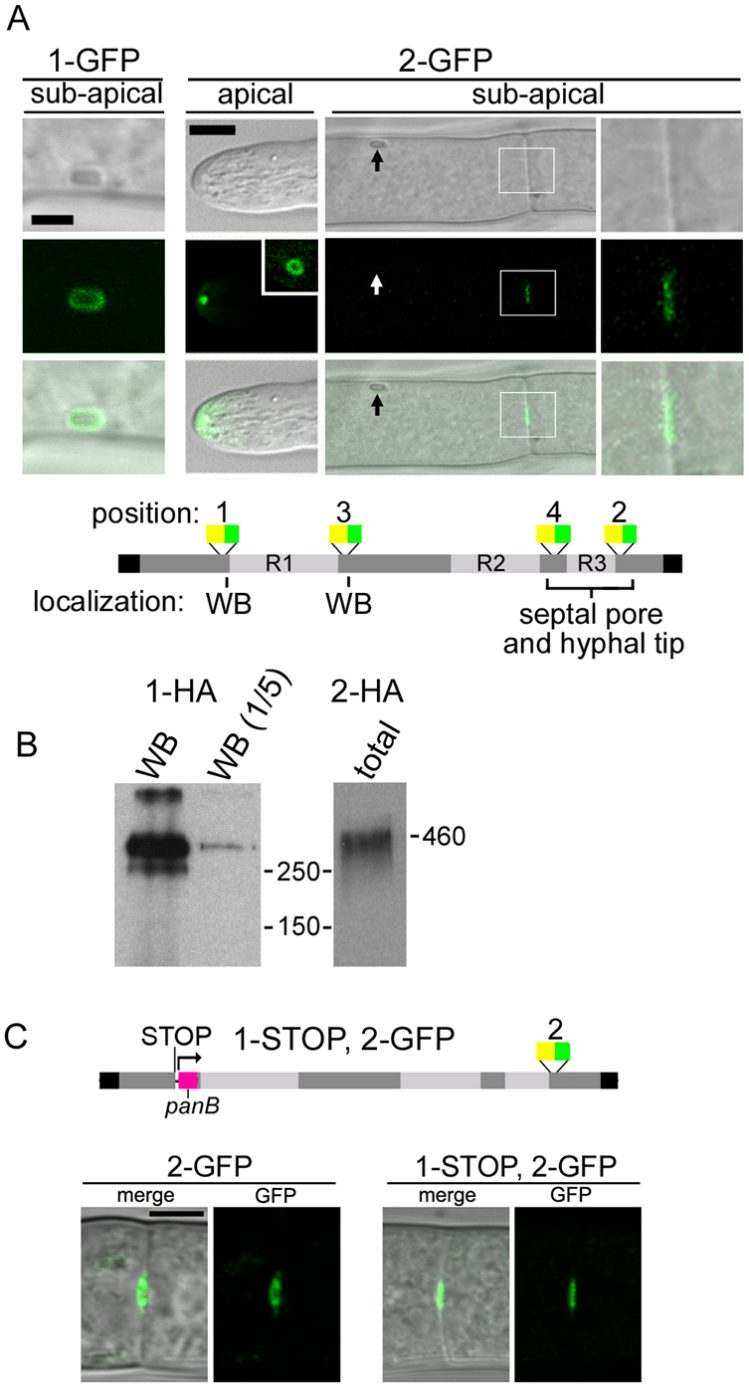
Marker fusion tagging (MFT) reveals distinct localization patterns of tagged versions of LAH; evidence for two transcription units. (A) Confocal microscopy reveals distinct non-overlapping localization patterns of MFTs at position 1 and 2. 1-GFP is localized exclusively to the WB (left panels) and 2-GFP is localized to the hyphal tip and septal pores (right panels). The inset shows 2-GFP ring structures, which can be seen in large hyphae. Arrow indicates the WB, which is not decorated by the 2-GFP tag. Box indicates the region that is magnified in the right most panels. Left bar = 2 µm, right bar = 5 µm. The lower cartoon summarizes results from MFT tags at positions 1, 2, 3 and 4. Yellow and green bars represent *hyg^r^* and *eGFP*, respectively. (B) HA epitope tags at positions 1 and 2 migrate as distinct polypeptides. 1-HA is detected in WB enriched fractions and 2-HA can be detected in total cell extracts. Molecular size in kDa is indicated. (C) The localization of 2-GFP is impervious to introduction of a stop codon at position 1, using the *panB* gene from *Aspergillus nidulans* as a selective marker (magenta bar). Lower panels show GFP localization in the indicated strains. Bar = 5 µm.

Using MFT, we integrated the *hyg^r^-gfp* cassette at two positions biased to the N- (1-GFP) and C- (2-GFP) terminus of the predicted *lah* gene; both of these strains display wild-type growth and WB segregation, indicating that the tagged proteins are functional (Figure 2). 1-GFP is localized to the WB surface in a pattern similar to that produced by the LAH^1–344^RFP protein (Figure 4A). Surprisingly, 2-GFP does not localize to the WB, but is found at the septal pore in sub-apical hyphae and in a single punctate structure immediately beneath the growing hyphal tip in apical compartments (Figure 4A), suggesting localization to the vicinity of the Spitzenkörper, a vesicle supply center tightly associated with hyphal growth [24],[25]. This punctate structure detached from the hyphal apex when hyphae stopped growing and in the largest hyphae, a LAH-2 ring structure with a diameter of up to 1 µm was observed (Figure 4A). Additional insertions biased to the predicted N- and C- terminus also localized either to the WB or the septal pore and hyphal tip (Figure 4A), further suggesting that *leashin* produces two distinct polypeptides. We next inserted the HA-epitope tag at positions 1 and 2 to estimate the size of *lah* encoded proteins (Figure 4B). 1-HA identifies an ∼400 kDa protein associated with WB enriched fractions while 2-HA encodes a polypeptide of ∼450 kDa, which can be detected in total cell extracts (Figure 4B). Together, these data suggest that the *lah* locus produces two distinct polypeptides localizing to different subcellular compartments.

*lah* can produce two proteins by distinct mechanisms; a single polypeptide can be post-translationally processed or alternatively, the locus may produce two independent transcripts. To distinguish these models, we subjected the 2-GFP strains to a second round of insertional mutagenesis and used the *Aspergillus nidulans panB* gene to insert an upstream stop codon at position 1 to produce 1-STOP, 2-GFP. If two proteins are processed from a single precursor, this should abolish the 2-GFP signals. Alternatively, if the pore-localized polypeptide is produced from a second transcriptional unit, 2-GFP should persist. Consistent with the later model, 2-GFP is readily detected at the septal pore in the presence of 1-STOP, suggesting that the 3′-end of *lah* encodes an independent gene (Figure 4C). We designated these two-transcription units *lah-1* and *lah-2*.

**Figure 5:**
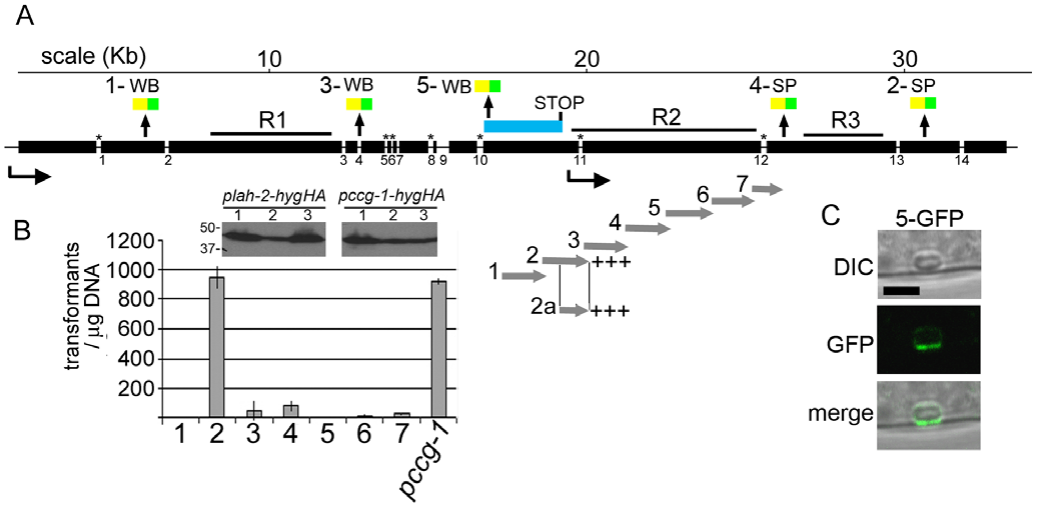
Molecular dissection of the *leashin* locus; identification of the 3′-end of *lah-1* and *lah-2* promoter. (A) The cartoon depicts the structure of the *lah* locus. Thick solid lines are exons and the position of MFT tags is indicated along with their localization to either the Woronin body (WB) or sepal pore (SP). Scale is indicated in kilobases (Kb). Introns are depicted as gaps and numbered. Introns identified in this study are marked with an asterisk. The alternative exon derived from splicing at intron 10 is indicated as a blue bar. (B) Gray arrows depict DNA fragments that were fused to the *hyg^r^* gene and transformed to assess their ability to promote Hygromycin resistance in a stable transformation assay. The graph indicates the transformation efficiency of these fragments compared with the strong *ccg-1* promoter. Inset panels show the levels of protein expressed from three randomly picked colonies expressing an epitope tagged version of *hyg^r^* from *plah-2* and *pccg-1* promoter sequences. (C) An MFT tag (5-GFP) in the alternatively spliced exon is localized to the WB with enrichment between the WB and cell cortex. Bar = 2 µm.

We next sought to define the *lah-2* promoter by fusing overlapping fragments from the putative promoter region to the *hyg^r^* gene and assessing their ability to confer Hygromycin resistance (Figure 5A and 5B). We assessed 7 fragments encompassing approximately 10 KB and found one fragment capable of engendering levels of stable Hygromycin resistant colonies comparable to the strong *ccg-1* promoter (Figure 5B) [26], suggesting that this region contains the *lah-2* promoter.

We were unable to detect *lah* transcripts by Northern blotting but were able to use RT-PCR to amplify overlapping fragments encompassing the entire *lah* locus (Figure S2). We confirmed all introns predicted at the Broad Institute *Neurospora crassa* database (http://www.broad.mit.edu/), and identified seven new introns (Figure 5A and Figure S2). Six of these are in-frame with predicted *lah* exons, suggesting that they do not significantly alter the encoded polypeptide. However, splicing of Intron 10, found immediately upstream of the *lah-2* promoter sequences produces a new reading frame and 2607 bp exon leading to a down-stream stop codon (Figure 5A). An MFT tag introduced into this frame decorates the WB, suggesting that these sequences encode the C-terminus of LAH-1. Moreover, unlike the N-terminally biased LAH-1 tag which uniformly decorates the WB (Figure 4A), the signal from this C-terminally biased tag is enriched between the WB and cell cortex (Figure 5C), suggesting that LAH-1 C-terminal sequences cluster at the cell cortex.

Collectively these data show *lah* encodes two large polypeptides from distinct regulatory sequences. LAH-1 is required for WB segregation and binds WBs via WSC and requires its C-terminal sequences for cortex association. LAH-2 and LAH-1 possess related repetitive sequences, but LAH-2 is not required for WB tethering and localizes in a novel pattern at both the growing hyphal apex and septal pore. Mutations in *lah-2* produce mild defects in maximum growth rate (Figure 2). We carefully examined the *lah-2* deletion mutant and found significant defects in radial growth early in colony establishment (Figure S3), suggesting that *lah-2* plays an important role in colony development.

**Figure 6:**
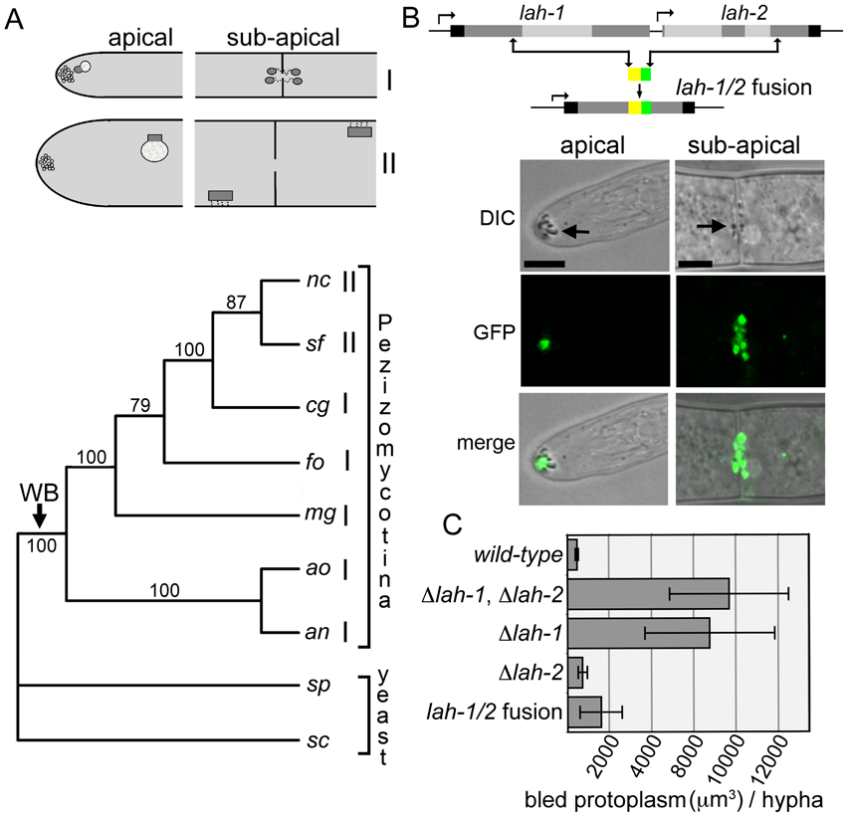
The ancestral pattern of WB localization can be produced in Neurospora by a LAH-1/LAH-2 fusion protein. (A) The schematic summarizes the two types of WB localization (I and II) in apical and sub-apical compartments. Woronin bodies are continuously formed *de novo* in the apical compartment (left panels) and are inherited into sub-apical compartments through different modes of cell cortex association (right panels). The lower panel shows a phylogenetic tree based on 18S r-RNA in species where WB localization has been determined. Arrow indicates the ancestor of the Pezizomycotina where WBs are presumed to have evolved. an, *Aspergillus nidulans* [15]; ao, *Aspergillus oryzae* [12]; cg, *Chaetomium globosum* [45]; mg, *Magnaporthe grisea* [11]; nc, *Neurospora crassa* [13], fo, *Fusarium oxysporum* [46]; sc, *Saccharomyces cerevisiae*; sp, *Schizosaccharomyces pombe*; sf, *Sordaria fimicola* (this study). (B) The cartoon shows the structure of the *lah* locus and MFT produced *lah-1/2* fusion. Panels show the localization of the LAH-1/2 fusion protein (GFP) and HEX assemblies (DIC), in apical and sub-apical compartments. Bars = 5 µm. (C) Woronin body function in the indicated strains was determined by measurement of protoplasmic bleeding.

Woronin bodies are synthesized in the apical hyphal compartment and segregated into sub-apical compartments by tethering to the cell cortex [13]–[15]. In most of the Pezizomycotina, mature organelles are tethered at the septal pore [8],[15]. By contrast, in Neurospora and close relative Sordaria, WB biogenesis is not closely associated with the hyphal tip and segregation is achieved by a dispersed pattern of cortex association [8],[13] (Figure 6A). To better understand the evolution of these two modes of cortex association, we used 18S ribosomal RNA to construct a phylogenetic tree from a group of species where Woronin body position has been determined and used the yeast *Saccharomyces cerevisiae* and *Schizosaccharomyces pombe*, which do not contain WBs or the *hex* gene [27] as outgroups. This tree produces a pattern of relationships consistent with previous reports [6],[7]. Neurospora and Sordaria comprise a clade within the Sordariomycetes and both possess the dispersed pattern of cortical association while all the basal lineages possess the septal-pore associated pattern, suggesting that septal pore tethering is the ancestral mode of segregation (Figure 6A).

Variations in the function of the *leashin* could determine evolutionary changes in patterns of cortical association. Specifically, we hypothesize that splitting of a single ancestral *lah* gene might have led to the derived pattern of WB-localization. In this case, the reunion of *lah-1* and *lah-2* should recreate the ancestral pattern of WB-distribution in Neurospora. MFT permits this experiment – we deleted intervening sequences, fusing N- terminal sequences of LAH-1 with C-terminal sequences of LAH-2 to produce a single chromosomally encoded polypeptide. Strains bearing this fusion as the sole version of *leashin* displays a pattern of WB-localization remarkably similar to the ancestral pattern (Figure 6B): HEX assemblies accumulate in the vicinity of the septal pore on both sides of the septum, and in a cluster immediately beneath the hyphal tip. Both of these localization patterns are abolished in a *wsc* deletion background (data not shown), indicating that the hybrid Leashin engages WBs via WSC. The LAH-1/2 expressing strain also grows slightly faster than the *lah-1* deletion strain (Figure S3) and presents a significant reduction in tip lysis induced protoplasmic bleeding (Figure 6C), suggesting that the fusion protein is partially functional.

## Discussion

Woronin body biogenesis occurs through a series of steps that link organelle morphogenesis and inheritance. HEX self-assembles to form the organellar core [10] and recruits WSC to the membrane. In turn, WSC envelops HEX assemblies in the matrix [14] and recruits LAH-1 in the cytoplasm (Figure 3). In the absence of LAH-1, nascent WBs fail to segregate and instead accumulate aberrantly in the apical compartment (Figure 1). Final fission of Woronin bodies from their mother peroxisomes also fails in the absence of LAH-1 (Figure 1B), suggesting that membrane fission [28] occurs after cortex association.

WB biogenesis has interesting parallels with the biogenesis of neuroendocrine dense-core secretory granules. In these organelles, aggregates of self-assembled core proteins are believed to promote budding from the trans-Golgi network through physical interaction with the membrane (for a review, see [29]). Aggregates at the center of dense-core secretory granules contain a complex mixture of proteins. By contrast, WBs with comparatively simple core composition and known membrane receptor [14] provide a genetically amenable system to study basic principles of aggregate promoted vesicle budding.

Neurospora LAH-1 possesses the properties expected of a tether; an N-terminal domain sufficient for WB localization (Figure 3) is linked to C-terminal sequences required for cell cortex association (Figure 2) by a poorly conserved linker domain enriched in repeat sequences (Figure S1). The central regions of fungal LAH proteins are not conserved at the level of primary sequence, but retain a similar character; they are highly acidic and enriched in the amino acids, PELS. Interestingly, PEVK repeats in the vertebrate muscle protein Titin adopt a random coiled configuration that forms an elastic filament [30]. WBs have been manipulated using laser-tweezers and observed recoiling towards the septum after being pulled and released [23], suggesting that WB-tethers are also elastic. In this case the similarities between PEVK regions of Titin and PELS regions of LAH proteins may represent overlapping convergent solutions to the problem of protein elasticity.

In most of the Pezizomycotina, WBs are associated with the septal pore at a distance of 100 to 200 nm and the length of the LAH proteins may determine the spacing between the cell cortex and WB. The largest known protein, vertebrate Titin is 4 mega-Daltons in size and has been purified and measured at approximately 1 µm in length [31]. *lah* genes in the Pezizomycotina are predicted to encode proteins between around 600 and 800 kDa and based on the size of Titin are expected to span a distance of around 200 nm, which is in reasonable agreement with the observed distance between WBs and the septum [15].

The presence of the WB specific genes *hex* [4],[10], *wsc* [14] and *leashin* in sequenced genomes of diverse members of the Pezizomycotina and their absence from sequenced genomes of fungi found outside this group, suggests that WBs arose in a common ancestor of the Pezizomycotina. Adaptations that occur at the origin of successful clades are likely to continue evolving as the group undergoes ecological specialization and evolutionary radiation. Neurospora and its close relative Sordaria are distinguished from other Pezizomycotina in several aspects of hyphal organization and physiology. In addition to the dispersed pattern of cortical association, both manifest large vegetative hyphae, extensive protoplasmic trafficking through septal pores [13],[32] (see Video S1 for trafficking in *Sordaria fimicola*) and unusually rapid growth, which in Neurospora can exceed 1 µm/second [33]. In nature, Neurospora occurs on burnt vegetation soon after forest fires [34] and this ecology may explain the evolution of rapid growth. We further speculate that extensive protoplasmic streaming may be incompatible with WB-tethering at the septal pore and provided a selective pressure for evolution of the delocalized pattern of cortex association.

**Figure 7:**
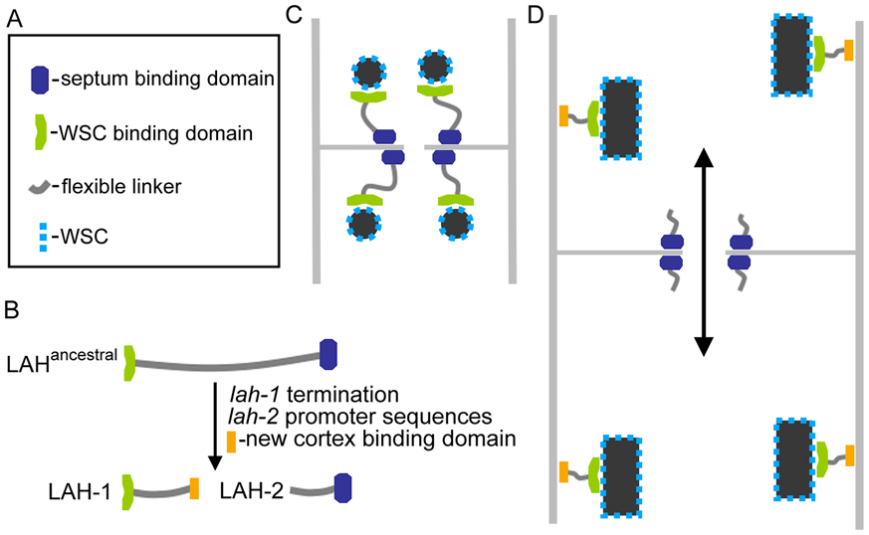
Model for the evolution of Woronin body tethering in the Pezizomycotina. (A) Legend indicates symbols used to depict domains of the Leashin tether. (B) The minimal events associated with splitting of the ancestral *leashin* locus are indicated. (C) Model for septal pore associated WB-tethering in most of the Pezizomycotina. (D) Model of WB tethering in Neurospora and Sordaria. The double-headed arrow indicates extensive protoplasmic streaming that can be observed in Neurospora and Sordaria (See Video S1).

Our analysis suggests that three events were required to evolve *lah-1* and *lah-2* from a single ancestral locus (Figure 7). These are - evolution of promoter sequences for independent production of *lah-2*, intragenic termination for the production of *lah-1* and acquisition of a new cortex-binding domain in *lah-1*. Splicing at intron 10 results in an alternative exon that terminates with a stop codon immediately upstream of the *lah-2* promoter region (Figure 5A). MFT tags in this exon are enriched between the WB and cell cortex (Figure 5C) and this region is required for WB inheritance (Figure 2), suggesting that C-terminal sequences of LAH-1 constitute a new cortex-binding domain.

Tethering is a fundamental eukaryotic strategy to control and coordinate the activities and spatial distribution of cellular organelles. For example, in the secretory pathway tethers control Golgi architecture and help determine the specificity of vesicle trafficking (for reviews, see [35],[36]). Tethers can also provide stable connections between functionally related organelles allowing for their efficient communication [37]. The Leashin tether promotes WB inheritance and holds the organelle in position until signals from cellular damage induce release, translocation to the septal pore and membrane resealing. Future work focused on these aspects of WB function should provide insights into reversible tethering, fungal signal transduction and plasma membrane dynamics.

## Materials and Methods

### Culture conditions, transformation, and microscopic methods

Vogel's N synthetic medium was used for growth in solid and liquid medium and for the induction of colonial growth; conidia were grown in plating medium [38]. *Neurospora crassa* conidia were transformed either by electroporation [39] or when cosmids were transformed, by chemical transformation of spheroplasts [40]. Strains used in this study can be found in Table S1. For the measurement of growth shown in Figure 2, Neurospora strains were grown on solid Vogel's medium overnight with appropriate supplements in 25 ml pipettes used as race tubes. Maximal growth rate was assessed after an initial period of 16 hours. Data shown are the mean and standard deviation from three replicates. Confocal microscopy and Woronin body quantification were conducted as previously described [14].

For the determination of protoplasmic bleeding (Figure 6C), strains were grown embedded in top agar containing sorbose, which induces tip lysis and colonial growth [38]. 0.75 mg/ml of Phyloxin dye is added to enhance the visualization of bled protoplasm. On the third day, images were obtained at the periphery of colonies using a stereomicroscope and the volume of the largest 15 bleeds from each colony was calculated using MetaVue software. A total of 225 individual hyphal bleeds were examined for each strain.

### Genetic methods

#### Identification and cloning of *leashin*

*leashin* was identified in a visual screen for mutants that accumulate WBs in the apical compartment. The original *leashin* mutant was spontaneous and unmarked, thus, linkage tester strains [41] were used to map the mutation to chromosome I and fine mapping with Caps markers [42] localized the mutation to the vicinity of contig 7. Cosmid clones encompassing this region were transformed into the *lah* mutant and H091 D4 [43], containing the majority of NCU02793 was found to complement the *lah* WB-segregation defect.

#### Generation of DNA fragments for homologous recombination at *leashin*

Fusion PCR was used to produce integration fragments for the truncation of chromosomally encoded *leashin*, and the integration of marker fusion tags (MFTs). Two MFT cassettes consisting of in-frame fusions of *Hyg^r^* to eGFP (GJP #1851) and 3× HA epitope tag (GJP #1873) were produced using the primers found in Table S2. For the production of fragments for integration by homologous recombination, three fragments are initially isolated using 6 primers, 1 and 2 amplify genomic DNA correspond to the left homologous flank, primers 3 and 4 amplify the selectable marker and primers 5 and 6 amplify the right homologous flank. Homologous regions were routinely designed between ∼500 to 700 bp in length. These three fragments were mixed and a fusion product was amplified using primers 1 and 6. These integration fragments were directly transformed into conidia using electroporation and selection at 100 mg/ml Hygromycin B. Integration at the correct position was verified by PCR using primers binding to DNA outside regions of homology (primers 7 and 8) and primers within the selectable marker. Because Neurospora conidia are multinucleate, primary transformants can be heterokaryons. Pure transformed strains were obtained through either sexual crosses or conidiation and identified using primers 9 and 10, which are designed to identify wild-type chromosomal loci. All of these primers can be found in Table S3. For *leashin* truncations (Figure 2), a stop codon was introduced into primers 2 and 3 and *hyg^r^* was expressed from the *trpC* promoter. Leashin deletion strains (Figure 6C and Figure S3) were produced using *hyg^r^* expressed from the *trpC* promoter and the primers found in Table S4. For the experiment shown in Figure 4C, we developed the *Aspergillus nidulans pan-2* homolog *panB*, AN1778.3, as a heterologous marker for the selection of transformants generated in the Neurospora *pan-2* mutant background. AN1778.3 was amplified from *Aspergillus nidulans* genomic DNA and flanking sequences from *leashin* were appended by fusion PCR and transformed as described above and selected for pantothenate prototrophy.

#### Identification of *lah-2* upstream activation sequences

We used fusion PCR to generate overlapping genomic DNA fragments fused to the *Hyg^r^* cassette. These fragments were transformed into wild-type conidia and assessed for their ability to produce stable Hygromycin resistant colonies. The strong *ccg-1* promoter was used as a control and primers used to generate these fragments can be found Table S5.

#### Mapping *leashin* introns by reverse transcriptase PCR (rt-PCR)

RNA was isolated from wild type and *Δlah-1*, *Δlah-2* deletion strain using the RNeasy Plant Mini Kit (Qiagen). Random primers were used to generate the cDNA library from total RNA using SuperScript lll First-strand synthesis system (Invitrogen) according to manufacturer's instructions. Primers (Table S6) were designed to amplify overlapping fragments (ranges from ∼400 to 1500 bp) of the entire predicted *leashin* gene. Wild-type genomic DNA served as a positive control and size differences between cDNA and genomic DNA indicated the presence of an intron. These fragments were cloned and sequenced to identify spliced sequences. RT-PCR using mRNA derived from a *leashin* deletion strain provided a control for primer specificity.

### Plasmids

Plasmids for the expression of WSC-eGFP (GJP#1081) and RFP-PTS1 (GJP#1406) were previously described [14]. To delete the C-terminal tail of WSC, two Xba I site encompassing the deleted region were created using site directed mutagenesis. The mutated plasmid was digested with Xba I and re-ligated resulting in the deletion of amino acids 236 to 307. To generate pmf272::^lah1–344^GFP, an Xba I to Pac I fragment encompassing the first 1032 nucleotides of *leashin* was inserted into pmf272 [26], to generate pmf272::^lah1–344^GFP (GJP#.1812). GFP was replaced using Eco RI and Pac I to create RFP (GJP#1898) and 3× HA (GJP#1848) tagged versions of this plasmid. Primers used for the construction of these plasmids can be found in Table S7.

### Cellular fractionation

For differential centrifugation, extracts were prepared from frozen Neurospora powder as previously described [13]. The lysate was passed through a 40-µm cell strainer to obtain a crude cellular extract. This extract was centrifuged at 100×g for 2 minutes to remove cellular debris. This cleared extract was centrifuged at 1 K×g and 10 K×g for 45 minutes and supernatant and pellet fractions were collected and analyzed by western blotting (Figure 3B). For Figure 4B (2-HA), 100 µl of the frozen Neurospora powder was added in equal volume of 2× loading buffer (2× LB), boiled and analyzed by western blotting. For Figure 4B (1-HA), frozen Neurospora powder were extracted in isolation buffer (20 mM Hepes pH 6.8, 150 mM KCL) with protease inhibitor cocktail (Roche) and a crude lysate was isolated as described above. The lysate was centrifuged at 6 K×g for 10 minutes and this pellet was re-suspended in isolation buffer containing 0.5% Triton X-100 (TX-100). The sample was passed through a 5-µm filter and centrifuged at 6 K×g for 10 minutes. The pellet was then washed twice in the same buffer using centrifugation at 8 K×g for 10 minutes. The final pellet, highly enriched in Woronin bodies was dissolved in 2× LB and analyzed by western blotting.

## Supporting Information

Figure S1 Sequence comparison with Dotter software [Sonnhammer EL, Durbin R] reveals repetitive Leashin sequences and shows that N- and C-termini tend to be conserved while intervening sequences are poorly conserved at the primary sequence level. an, *Aspergillus nidulans*; af, *Aspergillus fumigatus*; cg, *Chaetomium globosum*; nc, *Neurospora crassa*. Scale is in amino acid residues. [Sonnhammer EL, Durbin R (1995) A dot-matrix program with dynamic threshold control suited for genomic DNA and protein sequence analysis. Gene 167: GC1-10.]
(0.77 MB TIF)

Figure S2 Analysis of *leashin* mRNA by RT-PCR. 73 primer pairs were designed to amplify overlapping fragments covering the entire predicted *leashin* locus. First lane: Size standard. Second lane: RT-PCR using mRNA from a deletion of the entire predicted *lah* locus provides a negative control. Third lane: RT-PCR using Wild-type RNA as a template. Fourth lane: PCR using wild-type genomic DNA as a template. Size differences between lane 3 and 4 reveal introns. These fragments were cloned and sequenced and are indicated with a bar. Newly identified introns are marked with an asterisk. The box identifies a negative RT-PCR reaction produced by a primer pair falling within intron 9.
(1.31 MB TIF)

Figure S3 Growth rate of various *leashin* mutants. The indicated strains were grown on race tubes and the average growth rate over successive time periods was calculated.
(0.19 MB TIF)

Table S1 *Neurospora crassa* strains used in this study.
(0.07 MB PDF)

Table S2 Primers used to construct Hyg-HA and Hyg-GFP cassetes for MFT.
(0.03 MB PDF)

Table S3 Fusion PCR primers for integration of stop codons, MFT using Hyg-GFP and Hyg-HA and introduction of *Aspergillus nidulans pan-2* ortholog AN1778.3.
(0.05 MB PDF)

Table S4 Primers used to construct *Δlah1, Δlah2* and *Δlah1, Δlah2*.
(0.06 MB PDF)

Table S5 Primers used to identify *lah-2* promoter sequences.
(0.04 MB PDF)

Table S6 Primers used to map *leashin* by RT-PCR.
(0.04 MB PDF)

Table S7 Primers used to construct *lah1*-GFP/RFP/HA and *wsc-CΔ*.
(0.06 MB PDF)

Video S1 The translocation of protoplasm in *Sordaria fimicola*. This video encompasses approximately 10 seconds and shows the streaming of protoplasm through a septal pore at approximately 0.5 cm behind the growth front. Woronin bodies immobilized at the cell cortex are indicated with arrows.
(4.38 MB AVI)
