# Supplementary figures and images for: A Tether for Woronin Body Inheritance Is Associated with Evolutionary Variation in Organelle Positioning
Source: PLoS Genet. 2009 Jun 19;5(6):e1000521. doi: 10.1371/journal.pgen.1000521 (PMC2690989; doi:10.1371/journal.pgen.1000521)

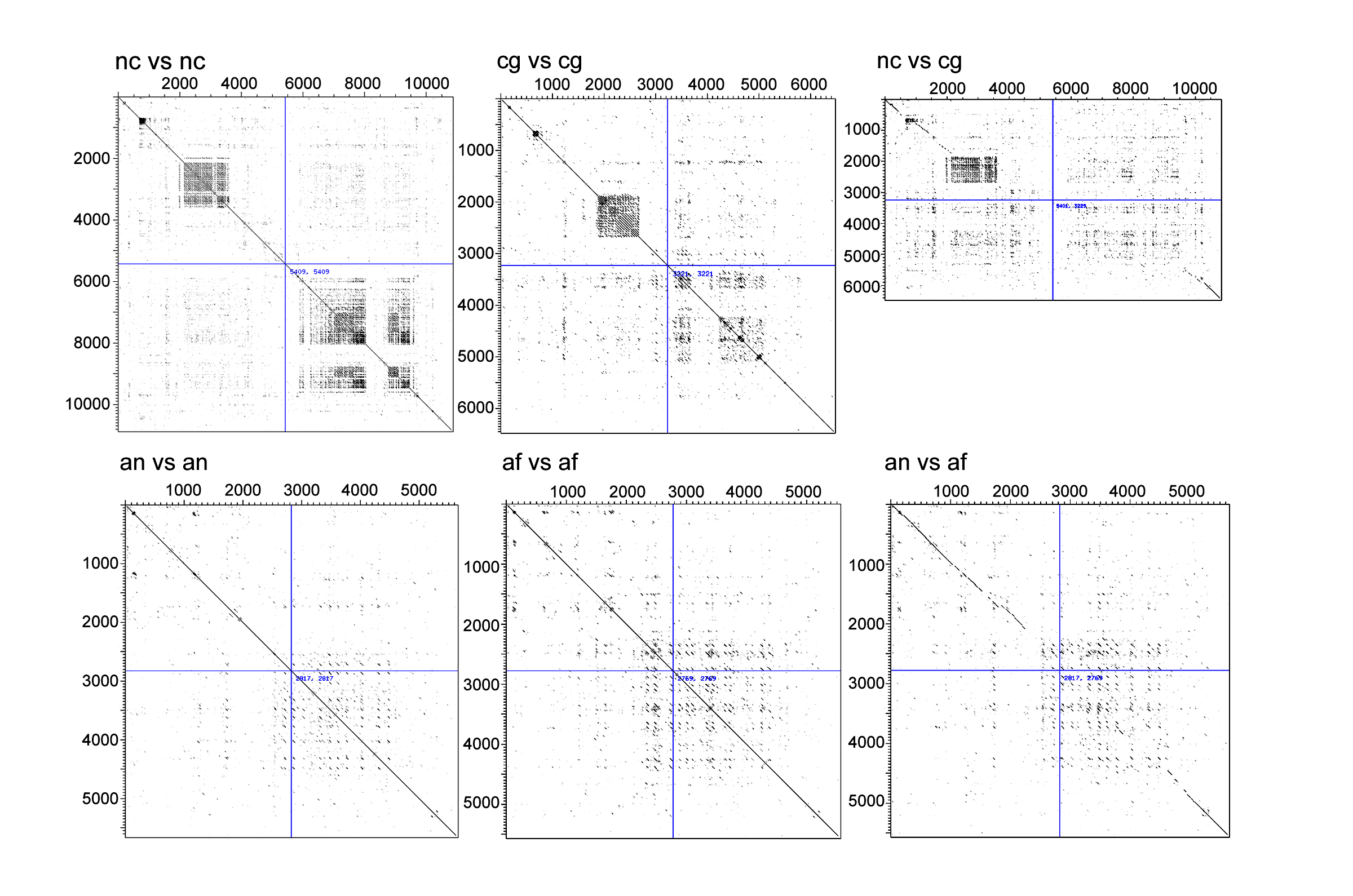

Supplement: Figure S1 — Sequence comparison with Dotter software [Sonnhammer EL, Durbin R] reveals repetitive Leashin sequences and shows that N- and C-termini tend to be conserved while intervening sequences are poorly conserved at the primary sequence level. an, Aspergillus nidulans; af, Aspergillus fumigatus; cg, Chaetomium globosum; nc, Neurospora crassa. Scale is in amino acid residues. [Sonnhammer EL, Durbin R (1995) A dot-matrix program with dynamic threshold control suited for genomic DNA and protein sequence analysis. Gene 167: GC1-10.] (0.77 MB TIF) [file pgen.1000521.s001.tif]

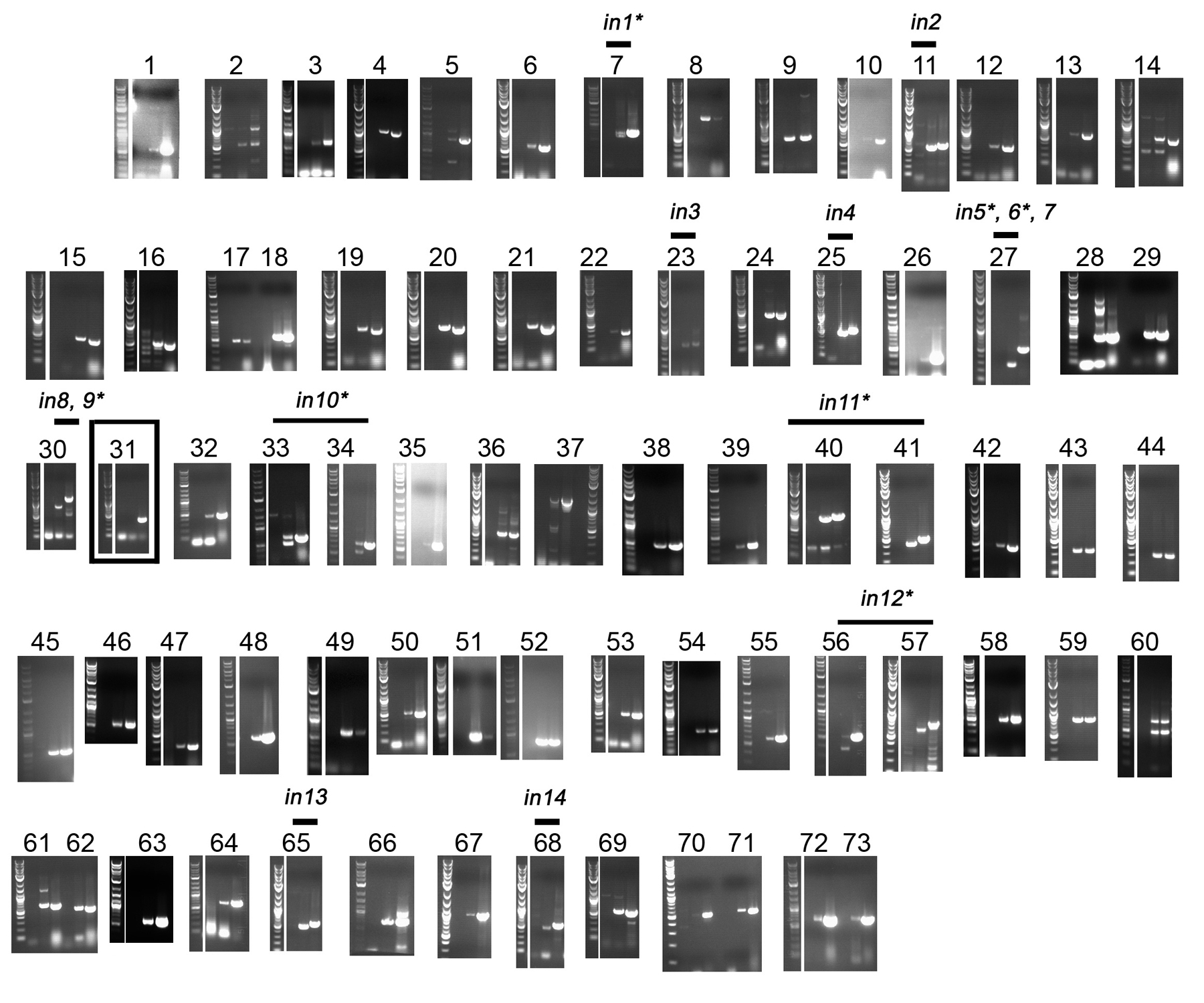

Supplement: Figure S2 — Analysis of leashin mRNA by RT-PCR. 73 primer pairs were designed to amplify overlapping fragments covering the entire predicted leashin locus. First lane: Size standard. Second lane: RT-PCR using mRNA from a deletion of the entire predicted lah locus provides a negative control. Third lane: RT-PCR using Wild-type RNA as a template. Fourth lane: PCR using wild-type genomic DNA as a template. Size differences between lane 3 and 4 reveal introns. These fragments were cloned and sequenced and are indicated with a bar. Newly identified introns are marked with an asterisk. The box identifies a negative RT-PCR reaction produced by a primer pair falling within intron 9. (1.31 MB TIF) [file pgen.1000521.s002.tif]

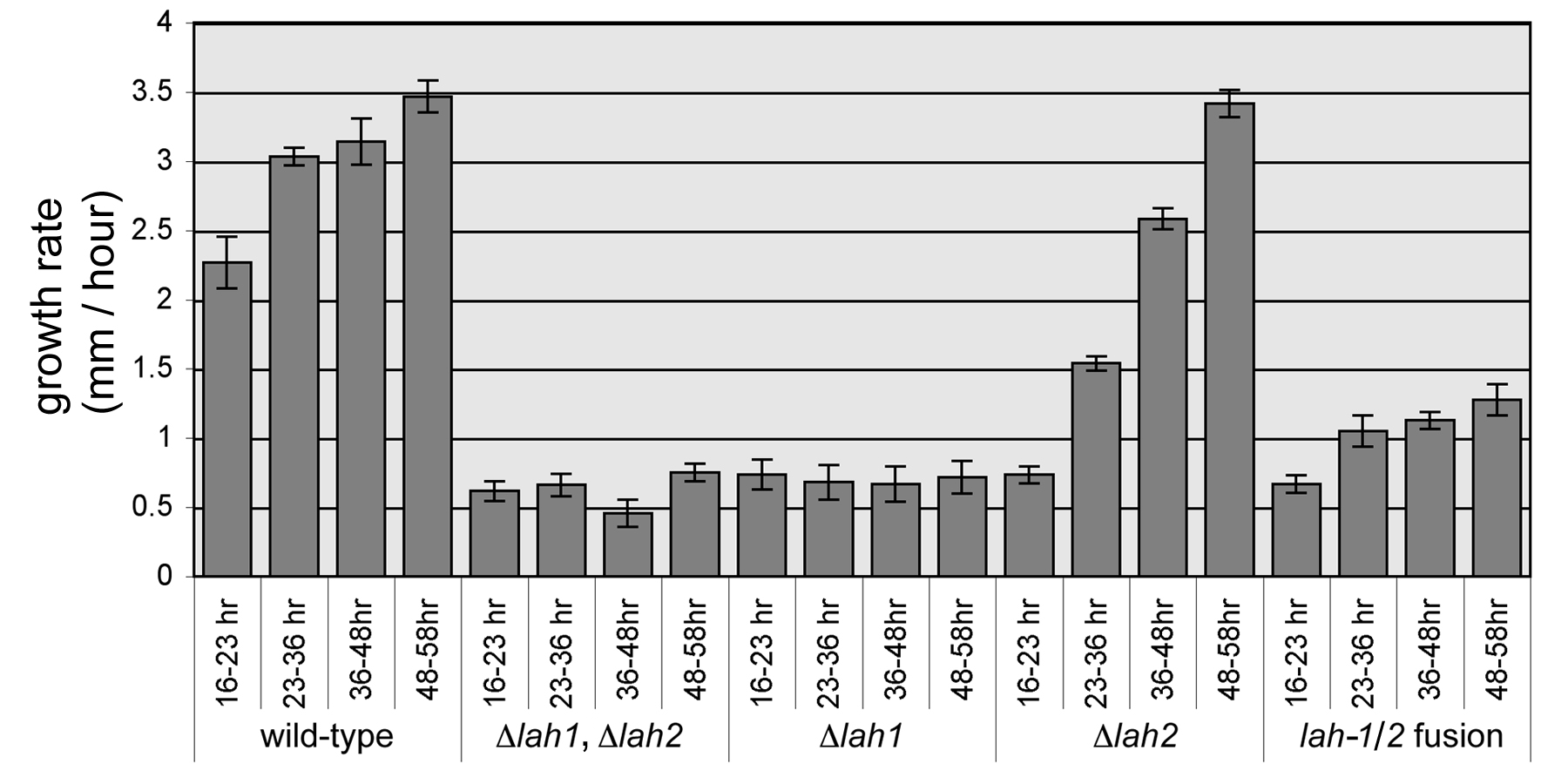

Supplement: Figure S3 — Growth rate of various leashin mutants. The indicated strains were grown on race tubes and the average growth rate over successive time periods was calculated. (0.19 MB TIF) [file pgen.1000521.s003.tif]
