# Supplementary material for: A Tether for Woronin Body Inheritance Is Associated with Evolutionary Variation in Organelle Positioning
Source: PLoS Genet. 2009 Jun 19;5(6):e1000521. doi: 10.1371/journal.pgen.1000521 (PMC2690989; doi:10.1371/journal.pgen.1000521)
Supplement: Table S1 — Neurospora crassa strains used in this study. (0.07 MB PDF) [file pgen.1000521.s004.pdf]

Table S1. *Neurospora crassa* strains used in this study

Strain	Genotype	Reference
GSF # 27	*mat A*	FGSC # 987
GSF # 71	*his-3-, Δmus 52::bar+, mat A*	FGSC # 9720
GSF # 59	*wsc-, mat A*	Liu (2008)
GSF # 269	*lah, pan-2-, mat a*	this study
GSF # 599	*lah, pan-2-, mat a* (GJP#961::WSC-eGFP, POK103::PHEX-RFP-PTS1)	this study
GSF # 1032	*his-3-, mat A* (GJP#961::WSC-eGFP, pMF272:: lah$^{1-344}$-RFP)	this study
GSF # 1082	*his-3-, mat A* (pMF272:: lah$^{1-344}$-HA)	this study
GSF # 1085	*Δwsc, his-3-, pan-2-, mat A* (pMF272:: lah$^{1-344}$-HA)	this study
GSF # 728	*Δwsc, his-3-, pan-2-, mat A* (GJP#961::wscΔC-eGFP, POK103::PHEX-RFP-PTS1)	this study
GSF #1092	*Δwsc, his-3-, pan-2-, mat A* (GJP#961::wscΔC-eGFP, pMF272:: lah$^{1-344}$-RFP)	this study
GSF # 1249	*lah::hyg 3604 stop, his-3-, Δmus52:: bar+, mat A*	this study
GSF # 1163	*lah::hyg 14902 stop, his-3-, Δmus52:: bar+, mat A*	this study
GSF # 1552	*lah::hyg 16981 stop, his-3-, Δmus52:: bar+, mat A*	this study
GSF # 1536	*lah::hyg 17680 stop, his-3-, Δmus52:: bar+, mat A*	this study
GSF # 1089	*lah::hyg 25786 stop, his-3-, Δmus52:: bar+, mat A*	this study
GSF # 1216	*lah::hyg 31603 stop, his-3-, Δmus52:: bar+, mat A*	this study
GSF # 1083	*1::hyg-eGFP, his-3-, Δmus52:: bar+, mat A*	this study
GSF # 1123	*2::hyg-eGFP, his-3-, Δmus52:: bar+, mat A*	this study
GSF# 1220	*3::hyg-eGFP, his-3-, Δmus52:: bar+, mat A*	this study
GSF # 1210	*4::hyg-eGFP, his-3-, Δmus52:: bar+, mat A*	this study
GSF # 1829	*5::hyg-eGFP, his-3-, Δmus52:: bar+, mat A*	this study
GSF # 1161	*1::hyg-HA, his-3-, Δmus52:: bar+, mat A*	this study
GSF # 1308	*2::hyg-HA, his-3-, Δmus52:: bar+, mat A*	this study
GSF # 1318	*1::pan-STOP, 2::hyg-eGFP, his-3-, pan-2-, Δmus52:: bar+, mat a*	this study
GSF # 1558, 1559 and 1560	*plah-2-hygHA, his-3-, Δmus52:: bar+, mat A*	this study
GSF # 1564, 1565 and 1566	*pccg-1-hygHA, his-3-, Δmus52:: bar+, mat A*	this study
GSF # 1140	*lah-1/2 eGFP fusion, his-3-, Δmus52:: bar+, mat A*	this study
GSF # 1211	*Δlah-1, Δlah-2, pan-2-, his-3-, Δmus52:: bar+, mat A*	this study
GSF # 1029	*Δlah-1, his-3-, Δmus52:: bar+, mat A* (pNEB193::*hygΔlah-1*)	this study
GSF# 1608	*Δlah-2, his-3-, Δmus52:: bar+, mat A*	this study
