# Supplementary material for: A Tether for Woronin Body Inheritance Is Associated with Evolutionary Variation in Organelle Positioning
Source: PLoS Genet. 2009 Jun 19;5(6):e1000521. doi: 10.1371/journal.pgen.1000521 (PMC2690989; doi:10.1371/journal.pgen.1000521)
Supplement: Table S2 — Primers used to construct Hyg-HA and Hyg-GFP cassetes for MFT. (0.03 MB PDF) [file pgen.1000521.s005.pdf]

Table S2. Primers used to construct Hyg-HA and Hyg-GFP cassetes for MFT

Construct	Sequence (5' to 3')
**Hyg-HA**	
SK101	GGTCATACCTTCTTAAGTTCG
SK102	GGAACATCGTAAGGGTATGCCATTTCCTTTGCCCTCGGACGAG
SK103	CTCGTCCGAGGGCAAAGGAAATGGCATACCCTTACGATGTTCC
SK104	GCTCGGACTGAGATAGGACG
**Hyg-GFP**	
hyg-gfp 1	TATTCTACCGAAGCATCGA
hyg-gfp 2	TCCTCGCCCTTGCTCACCATTTCCTTTGCCCTCGGACGAGT
hyg-gfp 3	ACTCGTCCGAGGGCAAAGGAAATGGTGAGCAAGGGCGAGGA
hyg-gfp 4	GCATGCTTACTTGTACAGCT
