# Supplementary material for: A Tether for Woronin Body Inheritance Is Associated with Evolutionary Variation in Organelle Positioning
Source: PLoS Genet. 2009 Jun 19;5(6):e1000521. doi: 10.1371/journal.pgen.1000521 (PMC2690989; doi:10.1371/journal.pgen.1000521)
Supplement: Table S3 — Fusion PCR primers for integration of stop codons, MFT using Hyg-GFP and Hyg-HA and introduction of Aspergillus nidulans pan-2 ortholog AN1778.3. (0.05 MB PDF) [file pgen.1000521.s006.pdf]

Table S3. Fusion PCR primers for integration of stop codons, MFT using Hyg-GFP and Hyg-HA and introduction of Aspergillus nidulans *pan-2 ortholog,* AN1778.3.

Hyg-Stop	Sequence (5' to 3')
**3604 stop**	
3604--1	GAGCCCAAGCCCAAGGTCGA
3604--2 stop	TGCTCCTTCAATATCATCTTTCAGCTCCGCTCACGTTCAGGTTC
3604--3 stop	GAACCTGAACGTGAGCGGAGCTGAAAGATGATATTGAAGGAGCA
3604--4 stop	CTCAGCGACCTTCTGAAGCACAAGTTCGGTCGGCATCTACTC
3604--5 stop	GAGTAGATGCCGACCGAACTTGTGCTTCAGAAGGTCGCTGAG
3604--6	TGCGCAGAATGGACTCCCACT
3604--7	CAACGAGCGCGACGTTCCGA
3604--8	CTGTTGTCCGGGTTGAATGTC
3604--9	GTTCGCGAATCCGAGCCACT
3604--10	GGGTAGCACTAGATTCAGCA
**14902 stop**	
14902--1	CTCATTCCTCCGGGGCCGCAG
14902--2 stop	TGCTCCTTCAATATCATCTTTCAGGTTCCCTCAGTATCAACGGA
14902--3 stop	TCCGTTGATACTGAGGGAACCTGAAAGATGATATTGAAGGAGCA
14902--4stop	GGCTTCGACGCTCCCGGGATCAAGTTCGGTCGGCATCTACTC
14902--5 stop	GAGTAGATGCCGACCGAACTTGATCCCGGGAGCGTCGAAGCC
14902--6	CGATCCAGTTCCCGGTCTCG
14902--7	CGTTGGCGATTCACCGACATC
14902--8	GGTCTCACCGGGAACTGCGAC
14902--9	GCGACAAGGCCAAAGACAAGC
14902--10	CTCGTCCTCGACAAGCCGCTC
**16981 stop**	
16981--1	GAGGACACGGGAGCCGACCAG
16981--2 stop	TGCTCCTTCAATATCATCTTTCAGCCGTTGCCTGATACGACCCG
16981--3 stop	CGGGTCGTATCAGGCAACGGCTGAAAGATGATATTGAAGGAGCA
16981--4stop	GTCGGCTCCGTCAAGTTGGCAAAGTTCGGTCGGCATCTACTC
16981--5 stop	GAGTAGATGCCGACCGAACTTTGCCAACTTGACGGAGCCGAC
16981--6	CGACTCCCTATACTCATCGCC
16981--7	CGCAAGATGAGCATGGGCTCG
16981--8	GCCTTGGCGCTCGTGCTCATC
16981--9	GTATCAGGCAACGGCCACCAG
16981--10	GTTGGCACTGGTCGTTGTCTC
**17680 stop**	
17680--1	CGTCAATGCAGCCACCACAGC
17680--2 stop	TGCTCCTTCAATATCATCTTTCATCGACGCTGTGTCTCCGACTG
17680--3 stop	CAGTCGGAGACACAGCGTCGATGAAAGATGATATTGAAGGAGCA
17680--4stop	CGATTCCAATTCGCCTTGGCGAAGTTCGGTCGGCATCTACTC
17680--5 stop	GAGTAGATGCCGACCGAACTTCGCCAAGGCGAATTGGAATCG
17680--6	CTGGACATCTTCAGGATTGAC
17680--7	CAGGCCCAGGAAGAGTATAAG
17680--8	GCGTGTCTGGGAGATCGCTGG
17680--9	CGAGCAGTCGGAGACACAGCG
17680--10	CACCCCCTTCGTGCCCTTCAG
**25786 stop**	
25786--1	CCTACTCACCGAGGCCCCAAC
25786--2 stop	TGCTCCTTCAATATCATCTTTCATGTCTGTTCGGCGGACGGAGAC
25786--3 stop	GTCTCCGTCCGCCGAACAGACATGAAAGATGATATTGAAGGAGCA
25786--4 stop	TAGTCTCTCCCAACGAATCCAAAGTTCGGTCGGCATCTACTC
25786--5 stop	GAGTAGATGCCGACCGAACTTTGGATTCGTTGGGAGAGACTA
25786--6	GGAGTATCTCGAGATGTCTCG
25786--7	CTTGATTCTATTGACGGTGC
25786--8	CAATATAACGTTGTAGGTTGG
25786--9	CATCAGTTAGAAACTCGTGGC
25786--10	CGGTCTGGATTTGGATCGGAC
**31603 stop**	
31603--1	GAGAGTTCCGAGGACAATGTG
31603--2 stop	TGCTCCTTCAATATCATCTTTCAGGCGACGGGCGTGGCAGATGT
31603-3 stop	ACATCTGCCACGCCCGTCGCCTGAAAGATGATATTGAAGGAGCA
31603--4stop	CAATCCACCATCCATCAGACAAGTTCGGTCGGCATCTACTC
31603--5 stop	GAGTAGATGCCGACCGAACTTGTCTGATGGATGGTGGATTG
31603--6	TAGACTAGCTAACAAGTTAGG
31603--7	GAACTACTGAGACTCCCAAG
31603--8	CCGTAAGAGGGTAGGGACTTG
31603--9	GCAGAGAAGGATGCCCAGATC
31603--10	GCTTGGACGACTGCTTCCGTG
Aspergillus Nidulans pan	
1--1	GAGCCCAAGCCCAAGGTCGA
1--2 an pan p	CGAATTAAATAGAGTAGTCTTTCAGCTCCGCTCACGTTCAGGTTCC
1--3 an pan p	GGAACCTGAACGTGAGCGGAGCTGAAAGACTACTCTATTTAATTCG
1--4 an pan	CTCAGCGACCTTCTGAAGCACTCATTTCTCTTCAGGTAATTC
1--5 an pan	GAATTACCTGAAGAGAAATGAGTGCTTCAGAAGGTCGCTGAG
1--6	TGCGCAGAATGGACTCCCACT
1--7	CAACGAGCGCGACGTTCCGA
1--8 an pan	GGATTGGCTTGGGCTGCTCCC
1--9 an pan	GGTTAAGAGCCGAGCGTATCC
1--10	CTGTTGTCCGGGTTGAATGTC
**Test for integreation site**	
hyg-f	CCTGCCTGAAACCGAACTGCC
hyg-r	GAGCTGCATCAGGTCGGAGAC

Hyg-GFP	Sequence (5' to 3')
**1-GFP**	
1--1	GAGCCCAAGCCCAAGGTCGA
1--2 gfp	CGGTGAGTTCAGGCTTTTTCATGCTCCGCTCACGTTCAGGTTC
1--3 gfp	GAACCTGAACGTGAGCGGAGCATGAAAAAGCCTGAACTCACCG
1--4 gfp	CTCAGCGACCTTCTGAAGCACCTTGTACAGCTCGTCCATGCCGA
1--5 gfp	TCGGCATGGACGAGCTGTACAAGGTGCTTCAGAAGGTCGCTGAG
1--6	TGCGCAGAATGGACTCCCACT
1--7	CAACGAGCGCGACGTTCCGA
1--8	CTGTTGTCCGGGTTGAATGTC
1--9	GTTCGCGAATCCGAGCCACT
1--10	GGGTAGCACTAGATTCAGCA
**2-GFP**	
2--1	GCGTCGCAATTGTGGCCGGCA
2--2 gfp	CGGTGAGTTCAGGCTTTTTCATCGGTAGACTGTTGTAAGAATGC
2--3 gfp	GCATTCTTACAACAGTCTACCGATGAAAAAGCCTGAACTCACCG
2--4 gfp	TGACAGGGCCAATGCTGCTTCTCTTGTACAGCTCGTCCATGCCGA
2--5 gfp	TCGGCATGGACGAGCTGTACAAGAGAAGCAGCATTGGCCCTGTCA
2--6 gfp	GTCGGCAAGGATGCTGGCCAGGT
2--7	CGTGATGATGGTAAGCAGTCCT
2--8	GTCTCCTCGAGTCGGTTGCAGGT
2--9	CGGATGACGACAGGAACAGGCA
2--10	GTGTACCAAGGAATGCCGACTT
**3-GFP**	
3--1	CCTTGCCTGACTCTCCTGCAG
3--2 gfp	CGGTGAGTTCAGGCTTTTTCATAGTAGCCTCGGAATCGCCCGC
3--3 gfp	GCGGGCGATTCCGAGGCTACTATGAAAAAGCCTGAACTCACCG
3--4 gfp	GGCCGAATTGCTCCTAAAGTTCTTGTACAGCTCGTCCATGCCGA
3--5 gfp	TCGGCATGGACGAGCTGTACAAGAACTTTAGGAGCAATTCGGCC
3--6	CTGCAGGAGAGTCAGGCAAGG
3--7	GGAAGCTGTTAAGGATATCAC
3--8	CCTTTCATCGACAGTGGTGGC
3--9	GGATGAAGAGGAGCAAGTTCC
3--10	CGTCCAGGTCGCCAAGCGTGC
**4-GFP**	
4--1	GCTGCTGCGATCGAGCGATCG
4--2 gfp	CGGTGAGTTCAGGCTTTTTCATTGTCTGTTCGGCGGACGGAGAC
4--3 gfp	GTCTCCGTCCGCCGAACAGACAATGAAAAAGCCTGAACTCACCG
4--4 gfp	GATAGTCTCTCCCAACGAATCCTTGTACAGCTCGTCCATGCCGA
4--5 gfp	TCGGCATGGACGAGCTGTACAAGGATTCGTTGGGAGAGACTATC
4--6	GGAGTGTCGTGATATTCCTCG
4--7	CTTGATTCTATTGACGGTGC
4--8	CAATATAACGTTGTAGGTTGG
4--9	CATCAGTTAGAAACTCGTGGC
4--10	CGGTCTGGATTTGGATCGGAC
**5-GFP**	
5--1	CAGGCGTCTTCCGGCCTTATG
5-2nif-gfp	GTGAGTTCAGGCTTTTTCATGCTCTTGTTGAGTGCGTTCGT
5-3nif-gfp	ACGAACGCACTCAACAAGAGCATGAAAAAGCCTGAACTCAC
5-4nifc-gfp	GACGTTGTCGATTGCGAAGAGCTTGTACAGCTCGTCCATGCCGA
5-5nifc-gfp	TCGGCATGGACGAGCTGTACAAGCTCTTCGCAATCGACAACGTC
5--6	ATGTCCGGCCGTTGTTGTGAG
5--7	GCTGCCGGCGATGCGTCTGG
5--10c	GATTCATTTTTGATGACGTTG
**1-2GFP**	
1--1	GAGCCCAAGCCCAAGGTCGA
1--2 gfp	CGGTGAGTTCAGGCTTTTTCATGCTCCGCTCACGTTCAGGTTC
1--3 gfp	GAACCTGAACGTGAGCGGAGCATGAAAAAGCCTGAACTCACCG
2--4 gfp	TGACAGGGCCAATGCTGCTTCTCTTGTACAGCTCGTCCATGCCGA
2--5 gfp	TCGGCATGGACGAGCTGTACAAGAGAAGCAGCATTGGCCCTGTCA
2--6 gfp	GTCGGCAAGGATGCTGGCCAGGT
1--7	CAACGAGCGCGACGTTCCGA
1--8	GTCTCCTCGAGTCGGTTGCAGGT
1--9	GTTCGCGAATCCGAGCCACT
2--10	GGGTAGCACTAGATTCAGCA
**Hyg-HA**	
**1-HA**	
1--1	GAGCCCAAGCCCAAGGTCGA
1--2 gfp	CGGTGAGTTCAGGCTTTTTCATGCTCCGCTCACGTTCAGGTTC
1--3 gfp	GAACCTGAACGTGAGCGGAGCATGAAAAAGCCTGAACTCACCG
1--4ha	CTCAGCGACCTTCTGAAGCACGGAAGCCATACCGGTATGAGC
1--5ha	GCTCATACCGGTATGGCTTCCGTGCTTCAGAAGGTCGCTGAG
1--6	TGCGCAGAATGGACTCCCACT
1--7	CAACGAGCGCGACGTTCCGA
1--8	CTGTTGTCCGGGTTGAATGTC
1--9	GTTCGCGAATCCGAGCCACT
1--10	GGGTAGCACTAGATTCAGCA
**2-HA**	
2--1	GCTGCTGCGATCGAGCGATCG
2--2 gfp	CGGTGAGTTCAGGCTTTTTCATTGTCTGTTCGGCGGACGGAGAC
2--3 gfp	GTCTCCGTCCGCCGAACAGACAATGAAAAAGCCTGAACTCACCG
2--4ha	GATAGTCTCTCCCAACGAATCGGAAGCCATACCGGTATGAGC
2--5ha	GCTCATACCGGTATGGCTTCCGATTCGTTGGGAGAGACTATC
2--6	GGAGTGTCGTGATATTCCTCG
2--7	CTTGATTCTATTGACGGTGC
2--8	CAATATAACGTTGTAGGTTGG
2--9	CATCAGTTAGAAACTCGTGGC
2--10	CGGTCTGGATTTGGATCGGAC
