# Supplementary material for: A Tether for Woronin Body Inheritance Is Associated with Evolutionary Variation in Organelle Positioning
Source: PLoS Genet. 2009 Jun 19;5(6):e1000521. doi: 10.1371/journal.pgen.1000521 (PMC2690989; doi:10.1371/journal.pgen.1000521)
Supplement: Table S4 — Primers used to construct Δlah1, Δlah2 and Δlah1, Δlah2. (0.06 MB PDF) [file pgen.1000521.s007.pdf]

Table S4. Primers used to construct *Δlah1, Δlah2D and Δlah-1, Δlah-2*

Δlah1	**Sequence (5' to 3')**
5’ UTR (sal1)f	CGGTCGACGCCTTCCTTGGGTTCG
2kb in orf (Ecor1)r	GGCGAATTCCTCGGCCAGAACATGGTTGG
STM not1-f 5'UTR	CATACATAGTGTTTAGTATCGCGGCCGCTTACCATACCATCCTAGCCG
STM not1-r 5'UTR	CGGCTAGGATGGTATGGTAAGCGGCCGCGATACTAAACACTATGTATG
STM pac1-f ORF	GACTCTGACCTTGCCTATGGTTAATTAACTCAATACTCGGACGAGAAG
STM pac1-r ORF	CTTCTCGTCCGAGTATTGAGTTAATTAACCATAGGCAAGGTCAGAGTC

Δlah2	**Sequence (5' to 3')**
test3-1	CGCTCTTCCATGTCTGGTGCT
test3-2	CGGTGAGTTCAGGCTTTTTCATATCATCCTCAAACTCATCATC
test3-3	GATGATGAGTTTGAGGATGATATGAAAAAGCCTGAACTCACCG
31603--4stop	CAATCCACCATCCATCAGACAAGTTCGGTCGGCATCTACTC
31603--5 stop	GAGTAGATGCCGACCGAACTTGTCTGATGGATGGTGGATTG
31603--6	TAGACTAGCTAACAAGTTAGG

Δlah-1, Δlah-2	**Sequence (5' to 3')**
Δlah-1	TCTGCACTAGTGTACCCAGG
Δlah-2	CGGTGAGTTCAGGCTTTTTCATTGTTGACCGCTTGGGTATAGG
Δlah-3	CCTATACCCAAGCGGTCAACAATGAAAAAGCCTGAACTCACCG
31603--4stop	CAATCCACCATCCATCAGACAAGTTCGGTCGGCATCTACTC
31603--5 stop	GAGTAGATGCCGACCGAACTTGTCTGATGGATGGTGGATTG
31603--6	TAGACTAGCTAACAAGTTAGG
