# Supplementary material for: A Tether for Woronin Body Inheritance Is Associated with Evolutionary Variation in Organelle Positioning
Source: PLoS Genet. 2009 Jun 19;5(6):e1000521. doi: 10.1371/journal.pgen.1000521 (PMC2690989; doi:10.1371/journal.pgen.1000521)
Supplement: Table S5 — Primers used to identify lah-2 promoter sequences. (0.04 MB PDF) [file pgen.1000521.s008.pdf]

Table S5. Primers used to identify *lah2* promoter sequences

Testing promoters	Sequence (5' to 3')
test1-1	GATCCCGGGAGCGTCGAAGCC
test1-2	CGGTGAGTTCAGGCTTTTTCATAGACCCGATGATGTTCCCGGT
test1-3	ACCGGGAACATCATCGGGTCTATGAAAAAGCCTGAACTCACCG
test2-1	CGAGCACTCGTCCCAGACGCA
test2-2	CGGTGAGTTCAGGCTTTTTCATAGAGCTTGTAGTGGCAGTGGC
test2-3	GCCACTGCCACTACAAGCTCTATGAAAAAGCCTGAACTCACCG
test2a-1	GCAGTCAATCCTGAAGATGTC
test2a-2	CGGTGAGTTCAGGCTTTTTCATAGAGCTTGTAGTGGCAGTGGC
test2a-3	GCCACTGCCACTACAAGCTCTATGAAAAAGCCTGAACTCACCG
test3-1	CGCTCTTCCATGTCTGGTGCT
test3-2	CGGTGAGTTCAGGCTTTTTCATATCATCCTCAAACTCATCATC
test3-3	GATGATGAGTTTGAGGATGATATGAAAAAGCCTGAACTCACCG
test4-1	CCAGAAAACATCGAGCTTCCG
test4-2	CGGTGAGTTCAGGCTTTTTCATCTGAACATGCGGAGGAATAAT
test4-3	ATTATTCCTCCGCATGTTCAGATGAAAAAGCCTGAACTCACCG
test5-1	CACAAGGAGGATGAGCTTCCT
test5-2	CGGTGAGTTCAGGCTTTTTCATCTCAATGTCCACTTGTTCCAC
test5-3	GTGGAACAAGTGGACATTGAGATGAAAAAGCCTGAACTCACCG
test6-1	CATATTGAGCGCTCCGCTTCG
test6-2	CGGTGAGTTCAGGCTTTTTCATGGGTTGTTTGCCCTTGCTGTC
test6-3	GACAGCAAGGGCAAACAACCCATGAAAAAGCCTGAACTCACCG
test7-1	GTCCTTCTACCTAAAGAAGTG
test7-2	CGGTGAGTTCAGGCTTTTTCATTGTCTGTTCGGCGGACGGAGA
test7-3	TCTCCGTCCGCCGAACAGACAATGAAAAAGCCTGAACTCACCG
ccg1-1	TAGAAGGAGCAGTCCATCTGC
ccg1-2	CGGTGAGTTCAGGCTTTTTCATTTTGGTTGATGTGAGGGGTTG
ccg1-3	CAACCCCTCACATCAACCAAAATGAAAAAGCCTGAACTCACCG
3' common primer	CAAATATATAGTCGCGTGGAG
**plah-2-hygHA**	
test2-1	CGAGCACTCGTCCCAGACGCA
test2-2	CGGTGAGTTCAGGCTTTTTCATAGAGCTTGTAGTGGCAGTGGC
test2-3	GCCACTGCCACTACAAGCTCTATGAAAAAGCCTGAACTCACCG
site2 hyg-ha -4	TGACATGGAGCTATTAAATCACTAAGCGTAATCTGGAACGTC
site2 hyg-ha -5	GACGTTCCAGATTACGCTTAGTGATTTAATAGCTCCATGTCA
3' common primer	CAAATATATAGTCGCGTGGAG
**pccg1-1-hygHA**	
ccg1-1	TAGAAGGAGCAGTCCATCTGC
ccg1-2	CGGTGAGTTCAGGCTTTTTCATTTTGGTTGATGTGAGGGGTTG
ccg1-3	CAACCCCTCACATCAACCAAAATGAAAAAGCCTGAACTCACCG
ccg1hyg-ha -4	TGACATGGAGCTATTAAATCACTAAGCGTAATCTGGAACGTC
ccg1 hyg-ha -5	GACGTTCCAGATTACGCTTAGTGATTTAATAGCTCCATGTCA
3' common primer	CAAATATATAGTCGCGTGGAG
