# Supplementary material for: A Tether for Woronin Body Inheritance Is Associated with Evolutionary Variation in Organelle Positioning
Source: PLoS Genet. 2009 Jun 19;5(6):e1000521. doi: 10.1371/journal.pgen.1000521 (PMC2690989; doi:10.1371/journal.pgen.1000521)
Supplement: Table S6 — Primers used to map leashin by RT-PCR. (0.04 MB PDF) [file pgen.1000521.s009.pdf]

Table S6. Primers used to map leashin by RT-PCR

No.	Forward	Sequence (5' to 3')	Reverse	Sequence (5' to 3')	Region	size (bp)
1	1f	GGCTCTAGAATGATTCCGCGAGACCAACGC	1r	TCGTGCTGAGGTTGCTCGTAC	1 - 521	521
2	2f	CGCCAACTCCAGCACTTCGT	2r	GGTAGGGCTGCGCAGGGTGT	165-700	535
3	3f	GAGGCGCCAGGAGAAGGAGCG	3r	CCGAGTCTGAAGCCGTATCCC	383 -1015	632
4	4f	CCAACTTCCGTCGCCAGAATC	4r	GGCGAATTCCTCGGCCAGAACATGGTTGG	896 - 2001	1105
5	5f	CGACCCATGACCTCGAGTTCG	5r	CACGGTAATCGGCTTCGTCACG	1802 - 2632	809
6	6f	GGCGCGCTTAGCACGTCTCTC	6r	GCACCTCGAGAAAGTCCGTCG	2499 - 3021	522
7	7f	GAGCCCAAGCCCAAGGTCGA	7r	TGCTCCTTCAATATCATCTTTCAGCTCCGCTCACGTTCAGGTTC	2896 - 3603	707
8	8f	GAGCCCAAGCCCAAGGTCGA	8r	GGGTAGCACTAGATTCAGCA	2896 - 4140	1244
9	9f	GTTCGCGAATCCGAGCCACT	9r	GGGTAGCACTAGATTCAGCA	3631 - 4140	509
10	10f	GTTTAGTTCGAGAGATCCATC	10r	GCTGCACCTCGGTCTTTGTTG	4080 - 4723	643
11	11f	CAAAGCCGGCTGACGTTGCTG	11r	CTGTTGTCCGGGTTGAATGTC	4679 - 5306	627
12	12f	CTGCGCAAAGCGGAAATTGG	12r	GTCCGAACCGGCGTTGCTGCC	5236 - 5775	539
13	13f	GGTTCGGACAGTGCCCCCGAG	13r	TCAAGACTCTCCATTTGGGG	5767 - 6578	811
14	14f	CCCCAAATGGAGAGTCTTGA	14r	GAGAGAGCGTCGTCCTCACC	6559 - 7127	568
15	15f	GTGGCCTTTCTTCGCCTACTC	15r	TTCCGGAACTGAAACCTCAC	7058 - 7539	481
16	16f	GGTCGTTGAGCGTGAGGTTTC	16r	GCTCAGGCACCTCCAACCCAC	7509 - 8023	514
17	17f	CAATACTTCTCCGGAGAGAAG	17r	GGCTTAGGCATAGACACCAG	7755 - 8234	479
18	18f	GATCGTTAAAGAACTGGTGTC	18r	CTCGTTGTCCGGAAGAGGTAG	8202 - 8775	573
19	19f	CTACCTCTTCCGGACAACGAG	19r	GGCTCGGGGATAGTGATTGAC	8755 - 9275	520
20	20f	CTCCTGTCCAAGAAGAGTTCG	20r	CTTGTGGCACCTCCTGGTAT	9155 - 9808	653
21	21f	GCTGTTTCTTTGCCAGTCGAG	21r	GTCCTCTTCAGCAACAGGGAC	9766 - 10413	647
22	22f	CCATCGCCAAGGAAGCTGTTAAG	22r	GAGAGCGGCGTGCCGCTTCTGC	10358 - 10697	339
23	23f	CGTCAAGGGTGACGAGGTGTC	23r	AGTAGCCTCGGAATCGCCCGC	10620 - 11286	666
24	24f	GCAGAAGCGGCACGCCGCTCTC	24r	CTGTGGAACTTGCTCCTCTTC	10676 - 11439	763
25	25f	GGATGAAGAGGAGCAAGTTCC	25r	CGTCCAGGTCGCCAAGCGTGC	11415 - 11952	537
26	26f	CAATGAGAGTAGCCAGCCCTG	26r	CTCCGGTCCGGTCTGCGACTC	11899 - 12296	397
27	27f	CTGATGAGTCGCAGACCGGAC	27r	GTATTTCTTCGGGGAGGGTGG	12271 - 12851	580
28	28f	GGATCCACGGCCAATGAAAAC	28r	CGATACTGTAGCTAGTTGCTC	12809 - 13321	491
29	29f	GACGCAGCAAGGTGAATCCCAG	29r	CCTCTGCATTGGAACCAGTC	13237 - 13727	490
30	30f	CTCATTCCTCCGGGGCCGCAG	30r	TGCTCCTTCAATATCATCTTTCAGGTTCCCTCAGTATCAACGGA	13686 - 14901	1215
31	31f	GGTCAAACAACTTTGTGAGC	31r	GTTGCTGATCCACCGTATGTG	14117- 14475	358
32	32f	CTCCCCTTCCGTTGATACTGAG	32r	CTCGTCCGCTGCTCCCGTCTC	14874 - 15345	471
33	33f	CAGTGACGGCAATTCCATCC	33r	CGTCCTTGCTGAAGCTGTTCCTC	15261 - 15758	497
34	34f	CATGGGCGTGCCTTACCGACG	34r	GCTTGTCCAAACTCTTCATGC	15366 - 16010	644
35	35f	GGCATGAAGAGTTTGGACAAG	35r	GATTCGCGGCGCTCCGCCATG	15989 - 16626	637
36	36f	GAGGACACGGGAGCCGACCAG	36r	TGCTCCTTCAATATCATCTTTCAGCCGTTGCCTGATACGACCCG	16408 - 16980	572
37	37f	GATCCCGGGAGCGTCGAAGCC	37r	CGGTGAGTTCAGGCTTTTTCATAGACCCGATGATGTTCCCGGT	16282 - 17802	1520
38	38f	GCGATTCCCTACTGGGATCTC	38r	GCTCACCGTGACCTCCTCAAC	17760 - 18282	522
39	39f	GCTGGGGGGATCGGTGTCAGC	39r	CGGTGAGTTCAGGCTTTTTCATAGGTAGTTCCACCTCCTCAGG	17854 - 18303	449
40	40f	GAGAACAAAGACGGCGATGAG	40r	CGGTGAGTTCAGGCTTTTTCATAGAGCTTGTAGTGGCAGTGGC	17986 - 19203	1217
41	41f	GCAGTCAATCCTGAAGATGTC	41r	CGACCCATGACCTCGAGTTCG	18574 - 19092	518
42	42f	CACACATCCCACTTTTCACG	42r	GTGATGAGGGAGATGGAGGA	18941 - 19372	431
43	43f	TACATGCCTTCCATCCGTGCT	43r	AAGTAAGTCGGAAGATTCTTT	19306 - 19629	320
44	44f	GCTTCCTTCTTTTCTTCCTGT	44r	AAGTAAGTCGGAAGATTCTTT	19339 - 19629	290
45	45f	CTTCCTCCATCTCCCTCATC	45r	GCCATTAGAACAGCAGGGGAC	19351 - 19874	523
46	46f	CTCATGCGCAACCGTCATCATC	46r	GTGGTGATTGGAAGGTCCTTG	19826 - 20384	558
47	47f	GCTGAACTCTCATCTGCACAC	47r	GCGGTAGTCGCTCATCGTCAG	20266 - 20575	309
48	48f	CTCTGACGATGAGCGACTACC	48r	CATCTTCAGGCAATTGAGCAG	20553 - 21196	643
49	49f	CTGCTGCTCAATTGCCTGAAG	49r	GTCAAAGGTGAGGAAGGAAGTTC	21173 - 21827	654
50	50f	GATTCGACCTGGGGTGAGGCTG	50r	GCCGAAGGTGAGGCAGGAGCC	21772 - 22304	532
51	51f	CGTCTAATACTGTTCCTGAGG	51r	CAGGCGTGAACTGTTCCAATG	22256 - 22774	518
52	52f	CTTCGCCATTGGAACAGTTCACG	52r	GAGGCCAGAAGTTCAGCGTTAG	22748 - 23492	744
53	53f	CCTAACGCTGAACTTCTGGC	53r	CTTCGGGTGCAATTACAGGT	23470 - 23950	480
54	54f	CAGGAGAATGTCAATCTTGAG	54r	CTGCGCAGATTGCTCAGGCTCG	23899 - 24396	497
55	55f	CAGTCGAGCCTGAGCAATCTG	55r	GCTTACTTGTTGGGGCCTCGG	24371 - 24979	608
56	56f	GATCTTTCCGACGTTCAAGAG	56r	CGAAGGTCCACATTGTCTTC	24601 - 25154	553
57	57f	CCTACTCACCGAGGCCCCAAC	57r	TGCTCCTTCAATATCATCTTTCATGTCTGTTCGGCGGACGGAGAC	24951 - 25785	834
58	58f	GTCTCCGTCCGCCGAACAGAC	58r	GAACGATAGTCTCTCCCAACG	25764 - 26266	502
59	59f	GAGTAGATGCCGACCGAACTTTGGATTCGTTGGGAGAGACTA	59r	GGAGTATCTCGAGATGTCTCG	26240 - 27074	834
60	60f	GAGCTCCCTTCCTCGCCTGAAC	60r	GAGGTTCCTCGGGGATTTTAGG	27028 - 27712	684
61	61f	CCTAAAATCCCCGAGGAACCTC	61r	GCGTCGTCATATCTTGCTCA	27691 - 28360	669
62	62f	GAGGCGGATGAGCAAGATATG	62r	CTTTGTGGCATCTGCCTCGGAG	28333 - 28884	551
63	63f	CTAGCTCCGAGGCAGATGCCAC	63r	CTGGGGCGTCACGAGACAATCC	28859 - 29467	608
64	64f	TCCGAGGCAGATGCCACAAAG	64r	CCATTCGTCGTCGTCTGCTGG	28864 - 29484	620
65	65f	GGATTGTCTCGTGACGCCCCA	65r	CCACGGCATCGGCGCTCCTTC	29446 - 30057	611
66	66f	GAAGGAGCGCCGATGCCGTG	66r	CTCAGGCACTGGTTCCTGCACC	30037 - 30705	668
67	67f	GAACTACTGAGACTCCCAAG	67r	GGCGACGGGCGTGGCAGATGT	30665 - 31602	937
68	68f	ACATCTGCCACGCCCGTCGCC	68r	GATCTGGGCATCCTTCTCTGC	31582 - 32170	588
69	69f	GCAGAGAAGGATGCCCAGATC	69r	GCTTGGACGACTGCTTCCGTG	32150 - 32733	583
70	70f	GATCGCGACACGGAAGCAGTC	70r	TCAAACCTGACTCCCCATCCC	32705 - 33235	530
71	71f	GATCGCGACACGGAAGCAGTC	71r	AATCCACCATCCATCAGACAA	32705 - +21bp	551
72	72f	GATCGCGACACGGAAGCAGTC	72r	GCTTCCTTCTTTTCTTCCTGT	32705 - +113bp	643
73	73f	GATCGCGACACGGAAGCAGTC	73r	AAGTAAGTCGGAAGATTCTTT	32705 - +167bp	697
