# Supplementary material for: A Tether for Woronin Body Inheritance Is Associated with Evolutionary Variation in Organelle Positioning
Source: PLoS Genet. 2009 Jun 19;5(6):e1000521. doi: 10.1371/journal.pgen.1000521 (PMC2690989; doi:10.1371/journal.pgen.1000521)
Supplement: Table S7 — Primers used to construct lah1-GFP/RFP/HA and wsc-CΔ. (0.06 MB PDF) [file pgen.1000521.s010.pdf]

Table S7. Primers used to construct *lah1*-GFP/RFP/HA and w*sc-CΔ*

**lah$^{1\text{-}344}$ GFP (GJP#1812)**	**Sequence (5' to 3')**
xba1-5'lah1500ORF	GGCTCTAGAATGATTCCGCGAGACCAACGC
pac1-3'lah1500ORF	GCCTTAATTAACGCACCAGCAAGCGTTCCCCA
pac1-stm344lahf	CGGACTCGGACTCGGACTCGTTAATTAACGCGGAAGAGGCCAGCGCTAG
pac1-stm344lahr	CTAGCGCTGGCCTCTTCCGCGTTAATTAACGAGTCCGAGTCCGAGTCCG

**lah$^{1\text{-}344}$ RFP/HA**	**Sequence (5' to 3')**
pac1-rfp,f	GGCTTAATTAACATGGTGAGCAAGGGCGAGGAG
ecor1-rfp.r	GGCGAATTCTTACTTGTACAGCTCGTCCAT
pac1-ha.f	GGCTTAATTAACATGGCATACCCTTACGATGTTCC
ecor1-ha.r	GCCGAATTCCTAAGCGTAATCTGGAACGTC

**wsc-CΔ**	Sequence (5' to 3')
3'-Mut-XbaI-1	CGTCGCCGAAGTGCTTTCTAGACAAAGCGGCGAGGCGCTTC
3'-Mut-XbaI-2	GAAGCGCCTCGCCGCTTTGTCTAGAAAGCACTTCGGCGACG
